# Cardio-Rheumatic Diseases: Inflammasomes Behaving Badly

**DOI:** 10.3390/ijms26083520

**Published:** 2025-04-09

**Authors:** Farah Issa, Marah Abdulla, Faizah D. Retnowati, Huda Al-Khawaga, Hanin Alhiraky, Khalid M. Al-Harbi, Amal Al-Haidose, Zaid H. Maayah, Atiyeh M. Abdallah

**Affiliations:** 1 Department of Biomedical Sciences, College of Health Sciences, QU Health Sector, Qatar University, Doha 2713, Qatar; fi1608332@student.qu.edu.qa (F.I.); ma1303726@student.qu.edu.qa (M.A.); fr2401815@student.qu.edu.qa (F.D.R.); ha1509230@student.qu.edu.qa (H.A.-K.); ha1601407@student.qu.edu.qa (H.A.); amalah@qu.edu.qa (A.A.-H.); 2 Department of Pediatric, College of Medicine, Taibah University, Madinah 41477, Saudi Arabia; kalharbi@taibahu.edu.sa; 3 Department of Pharmaceutical Sciences, College of Pharmacy, QU Health Sector, Qatar University, Doha 2713, Qatar; almaayah@qu.edu.qa

**Keywords:** cardiovascular disease, inflammasome, NLRP3, caspase, Kawasaki disease, rheumatic heart disease

## Abstract

Cardio-rheumatology is an evolving and interdisciplinary field lying at the intersection of rheumatology and cardiovascular medicine that recognizes that individuals with autoimmune and inflammatory rheumatic complications have a much higher likelihood of developing cardiovascular diseases (CVDs). Inflammasomes are multiprotein complexes stimulated by the immune system after the detection of pathogens or cellular injury. Inflammasomes undergo a two-stage activation process initiated by nuclear factor (NF)-κB, subsequently playing a crucial role in innate immunity through activation of caspase 1 and the consequent release of proinflammatory cytokines such as IL-18 and IL-1β. However, a loss of control of inflammasome activation can cause inflammatory diseases in humans. Recent studies have focused on the role of inflammasomes in inflammatory cascades implicated in the pathogenesis of several diseases. Here, we review inflammasome activation, its mechanism of action, and its role in CVD. In particular, we describe the role of inflammasomes in rheumatic heart disease, Kawasaki disease, familial Mediterranean fever, ankylosing spondylitis, and rheumatoid arthritis as exemplars to illustrate pathobiological mechanisms and the potential for targeting inflammasomes for therapeutic benefit.

## 1. Introduction

Cardio-rheumatology is an evolving and interdisciplinary field lying at the intersection of rheumatology and cardiovascular medicine that recognizes that individuals with autoimmune and inflammatory rheumatic complications have a much higher likelihood of developing cardiovascular diseases (CVDs) [1]. Weber et al. recently described cardio-rheumatology as “a subspecialty that focuses on the impact of inflammation on the cardiovascular system” and the understanding of the “anti-inflammatory strategies encompassing both the treatment and potential complications of targeting inflammation” in those patients [2]. Reflecting this relationship between inflammation and CVD, rheumatic conditions such as systemic lupus erythematosus (SLE) and rheumatoid arthritis (RA) represent significant risk factors for the development of various cardiovascular conditions, including arrhythmias, heart failure, valvular heart disease, and coronary artery disease [3] and patients with autoimmune diseases have a three-fold increased risk of CVD [4]. This relationship between the two conditions highlights the importance of studying inflammatory pathways in cardiovascular diseases, which encompass a broad spectrum of conditions impacting the heart and blood vessels. The most common type of CVD is coronary artery disease (CAD), which is caused by the development of atherosclerotic plaques in the coronary arteries, which reduce blood flow to the myocardium [5] and, if untreated, can lead to myocardial infarction (MI), angina, and possibly heart failure, which clinically leads to exhaustion, dyspnea, and edema [2]. The complex spectrum of CVDs also includes arrhythmias and valvular heart disorders, each posing particular challenges for diagnosis and treatment, especially in the pediatric population [6].

**Figure 1:**
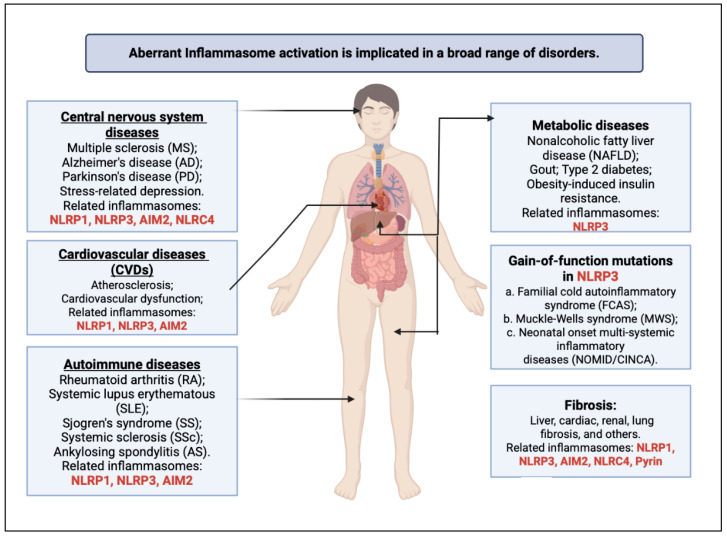
A summary of the multisystem impacts of inflammasome activation [10,11,12].

Although the relationship between CVD and inflammation is complex, elevated pro-inflammatory cytokines, autoantibodies, and immune complexes are known to create a pro-atherogenic environment that leads to endothelial dysfunction, a procoagulant state, and vascular inflammation [7]. Inflammatory responses are multifactorial, triggered by many factors, including pathogens and underlying genetic factors. As part of host inflammatory responses, inflammasomes represent a critical component of the innate immune system, functioning as multiprotein complexes that detect pathogenic microorganisms and stress signals, ultimately activating inflammatory responses [8]. Moreover, inflammasomes are being activated by pathogen-associated molecular patterns (PAMPs) and damage-associated molecular patterns (DAMPs), these complexes are essential for host defenses because they promote the production and release of pro-inflammatory cytokines like interleukin-18 (IL-18) and interleukin-1β (IL-1β) [9]. Understanding the action and interactions of inflammasomes is, therefore, vital for advancing new solutions to the management of inflammatory conditions and their cardiovascular consequences, such as in RHD, Kawasaki disease, familial Mediterranean fever, and RA, in which inflammation is known to act as a critical pathogenic link between rheumatic diseases and cardiovascular dysfunction (Figure 1, [10,11,12]) [13].

The complex mechanisms governing inflammasomes in CVD have emerged as a crucial area of research [14]. Inflammasomes have dual activity: they are protective in the context of infection but detrimental when dysregulated, underscoring the complexity of their role in cardiovascular health and disease. This complexity is particularly pronounced in cardio-rheumatology, where inflammasome activity affects the pathogenesis of different rheumatic heart diseases [15]. Inflammasomes, specifically the NLRP3 inflammasome produced by endothelial cells and macrophages, recognize oxidized low-density lipoprotein (oxLDL) particles, initiating an inflammatory cascade that contributes to plaque formation and instability. Furthermore, in MI, inflammasome activation is important for tissue repair but potentially exacerbates ischemic injury if not strictly controlled [12]. An additional significant risk factor for CVD, hypertension, is also associated with inflammasome activity, with high blood pressure causing arterial stiffness and endothelial dysfunction, consequent mechanical stress, the release of reactive oxygen species (ROS), and inflammasome activation [16].

Despite progress in the understanding inflammasome biology and its relationship with CVD, there is still a need to determine the details of inflammasome signaling pathways in CVD, ultimately paving the way for the development of targeted therapies that harness the protective aspects of inflammasomes while mitigating their deleterious effects. Understanding this need, here we aim to review the intricate relationship between inflammasomes and cardio-rheumatic diseases, elucidating how dysregulation in inflammasome signaling pathways contributes to the pathogenesis and progression of these conditions and how these pathways may be exploited to improve clinical outcomes.

## 2. Inflammasome Structure and Activation Pathways

Inflammasomes are multi-protein complexes found in the cytosol and widely expressed in many different cell types [17,18]. Inflammasomes play a vital role in the innate immune response, which acts as a first line of host defense that triggers adaptive immunity [19]. They are specifically involved in inflammatory responses occurring after cells sense or detect PAMPs or DAMPs [20].

All known inflammasomes consist of three common segments: a sensor (a pattern recognition receptor (PRR) to sense PAMPs and DAMPs); an adaptor (an apoptosis-associated speck-like protein, ASC), which includes a caspase activation and recruitment domain (CARD) and a pyrin domain (PYD); and an effector (pro-caspase 1) [21,22]. The CARD is responsible for linking the sensor with the effector, pro-caspase-1 [17]. Inflammasomes are assembled in several different cells, but primarily in macrophages and in other immune cells like dendritic cells and neutrophils [23].

The PRR, when present, determines the type of inflammasome: nucleotide-binding domain-like receptor (NLR), absent in melanoma 2-like receptor (ALR), or pyrin [24]. The NLR family, members of which are intracellular surveillance receptors, can be further divided into NLRP1, NLRP3, and NLCR4 [25]. Of all inflammasomes, the best-characterized inflammasome is NLRP3, mainly due to its role in the pathogenesis of inflammatory diseases [26]. NLRP3 inflammasomes are the main inflammasomes implicated in inflammatory and cardiovascular diseases [27]. The inflammasome pathway has two main steps: priming and activation [28].

### 2.1. Signal 1: Priming

The priming step is when the proteins are synthesized after transcription, which then wait for the other stimuli and proteolytic activation to assemble. Priming starts once PRRs, such as toll-like receptors (TLRs) found on the membranes of innate immune cells, recognize PAMPs released from microbial infections or DAMPs released from damaged or dying cells. It can also start after detecting the presence of tumor necrosis factor-alpha (TNF-α) [29]. Then, TLRs or the TNF receptor (TNF-R) induce the production of the transcription factor NF-κB by phosphorylating and lysing its inhibitor [30]. NF-κB then relocates to the nucleus and, using signaling molecules MyD88 and TRIF, upregulates the production of NLRP3 (usually present at low concentrations unable to initiate activation under steady-state conditions) and pro-IL-1β [31]. Recent studies have reported that caspase 8 and FADD, both apoptotic signaling molecules, are also needed for NLRP3 priming [32]. Priming also controls the modification of NLRP3 post-transcriptionally by ubiquitination and phosphorylation, which regulate the activation process [31,33].

### 2.2. Signal 2: Activation

**Figure 2:**
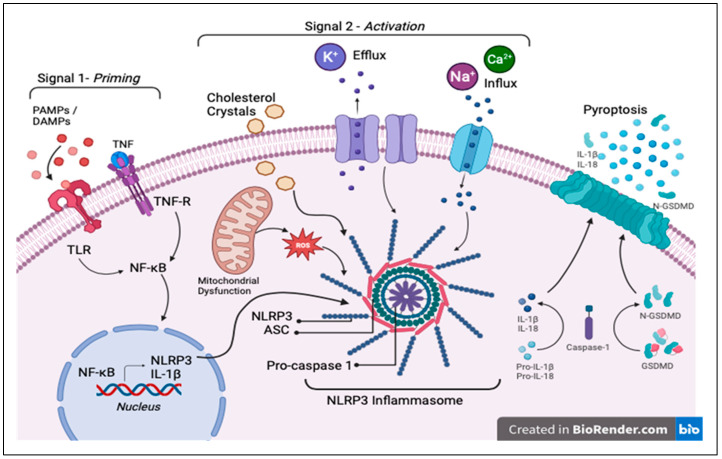
Summary of the signals controlling inflammasome activation, leading to pyroptosis.

The second signal, activation, requires cleavage and assembly, and is initiated by cell stressors or danger signals. For the NLRP3 inflammasome, NLRP3 ligands do not directly activate the inflammasome, though several triggers have been shown to play a role in its activation (Figure 2). One trigger is potassium (K^+^) efflux, with several NLRP3 activation stimuli triggering potassium release from the cell, decreasing intracellular potassium levels, and in turn, activating NLRP3 [34,35]. Another trigger is ion channel dysfunction, such as of P2X7, which results in an ion imbalance and influx of calcium (Ca^2+^) from the extracellular space, thereby activating inflammasomes [36]. Lysosomal damage is another trigger, as lysosomes cleave the components of the inflammasome and rupture, releasing their contents into the cytosol and promoting inflammasome assembly and activation [37]. Moreover, mitochondrial dysfunction activates NLRP3 inflammasomes [38,39] through the release of mitochondrial DNA and ROS into the cytosol [40,41].

Pro-caspase 1 bound to the inflammasome facilitates its own autocatalytic cleavage to form caspase 1, a crucial proteolytic enzyme in human hemostasis [30]. Activated caspase 1 cleaves the inactive forms of inflammatory cytokines pro-IL-1β and pro-IL-18 to produce mature and active forms of IL-1β and IL-18, which are downstream cytokine effectors of inflammasomes [42,43,44]. IL-1β is associated with several immune reactions that include the recruitment of inflammatory cells to sites of infection [45] and the production of genes that result in fever, hypotension, and vasodilation [31]. IL-18 induces vascular components of inflammation, increasing the release of cell adhesion proteins and the production of interferon (IFN-α) and several chemokines to enhance the cytosolic activity of natural killer (NK) cells [24]. IL-18 also contributes to the polarization of type 1 T-helper cells. Caspase 1 cleaves and activates gasdermin D, a membrane pore-forming protein [46,47], which translocates to the cell membrane to allow the N-terminal domain to form pores, activating lysis and causing the cells to die in an inflammatory form of programmed cell death known as pyroptosis, while also releasing intracellular contents along with IL-1β and IL-18 through these pores [14,48].

### 2.3. Other Inflammasome Pathways

In addition to NLRP3 inflammasomes, other types of inflammasomes are also involved in some cardio-rheumatic diseases, including AIM2, NLRP1, and NLRC4 inflammasomes [25]. These inflammasomes have distinct stimulation signals but ultimately culminate on the same inflammatory cascade that results in caspase 1 activation [27]. These inflammasomes differ in terms of the structure of the sensors. For example, AIM2 inflammasomes sense cytosolic DNA using their C-terminal domain to recruit procaspase 1 with the ASC to create the complex AIM2-ASC-pro-caspase-1 [49]. NLRP1 contains a C-terminal CARD and N-terminal PYD domains that interact with pro-caspase 1 directly without the need for the ASC adapter, although connecting to the ASC adapter can enhance NLRP1-mediated caspase 1 activation [50]. NLRC4 only has a CARD domain that directly recruits pro-caspase 1 in the absence of the ASC domain to form the NLRC4 inflammasome [14].

## 3. The Role of Inflammasomes in Cardio-Rheumatic Diseases

A pressing clinical challenge emerges from the recognition that inflammation not only causes acute CVD but also perpetuates chronic pathological alterations in CVDs. The therapeutic prospect of modulating inflammasomes in CVD therapy is underscored by the clinical endorsement of biologics targeting IL-1β for specific inflammatory conditions and the encouraging preclinical findings of various small molecule inflammasome inhibitors [51]. We therefore review selected CVDs associated with inflammasome activation to highlight the role of inflammasomes in cardio-rheumatic diseases (Table 1).

### 3.1. Familial Mediterranean Fever

The multisystem hereditary disease known as familial Mediterranean fever (FMF) affects the lungs, abdomen, and joints. People of Mediterranean heritage, including Arabs, Turks, Armenians, and Jews, are primarily affected by FMF, which is characterised by repeated episodes of inflammation, fever, peritonitis, synovitis, and pleuritis [52]. Since FMF symptoms usually start in childhood and frequently resemble those of other pediatric illnesses, genetic testing is highly suspected [53]. The regulatory function of pyrin in the inflammasome complex is disrupted by mutations in the MEFV gene, which codes for pyrin. This leads to unchecked inflammasome activation and an overabundance of pro-inflammatory IL-1β [54].

The symptoms of FMF are caused by this imbalance between pro-inflammatory and anti-inflammatory homeostasis. The prevalence of cardiac problems, particularly pericarditis, in FMF emphasises the necessity of close observation to avoid serious consequences [55]. For FMF patients, IL-1β blocking medication has been shown to be successful in lowering symptom frequency and enhancing quality of life [56]. Notably, this medication does more than only control the generation of IL-1β; it also effectively regulates the activation of inflammasomes [53].

### 3.2. Rheumatic Heart Disease

Rheumatic heart disease (RHD) is a serious cardiac condition that results from an autoimmune response to group A *Streptococcus* (GAS) infection, often originating in the throat or skin. RHD manifests as fatigue, chest pain, and shortness of breath, with pulmonary hypertension and heart failure. Acute rheumatic fever (ARF), the precursor to RHD, is a nonsuppurative, immune-mediated consequence of group A streptococcal pharyngitis (strep throat) characterized by multisystemic inflammation, including in the joints, skin, brain, and heart [57]. The disease predominantly affects children and young adults. The body’s immune system, in trying to combat GAS, produces antibodies that mistakenly target host tissues, particularly the heart valves, leading to inflammation, scarring, and eventual dysfunction. This process ultimately culminates in RHD [58]. The distinctive aspect of pediatric RHD for GAS infection is the increased vulnerability of the developing immune system, which might lead to an exaggerated autoimmune response to GAS infections. This response is highly likely to cause severe and recurrent cardiac damage, emphasizing the high demand for early detection and management to prevent RHD in children.

Inflammasomes, particularly the NLRP3 inflammasome, are essential to the immunological reaction seen in RHD. As noted above, when inflammasomes identify pathogenic microorganisms and stress signals, they activate caspase-1, which cleaves IL-1β and IL-18 into their active forms [24] to trigger a strong proinflammatory response that helps to remove pathogens by attracting immune cells to the infection site [59]. This maladaptive response is especially pronounced in pediatric RHD, where the developing immune system’s reaction can exacerbate heart valve damage. By encouraging a hyper-inflammatory state, autoantibodies are generated, and immune cell infiltration into the heart valves is promoted through proinflammatory enzymes [60]. This leads to acute inflammation, chronic scarring, and, over time, heart valve deformation with recurrent episodes of ARF, which are characteristic features of RHD [61,62]. Recurrent GAS infections make the situation even more unstable, as inflammasomes are reactivated by every subsequent infection, which feeds a vicious cycle of inflammation and immune-mediated damage [63]. Moreover, persistent inflammasome activation can lead to a chronic inflammatory state, which may have systemic implications and contribute to the emergence of additional comorbidities and a general deterioration in well-being [64,65].

The intricate involvement of inflammasomes in RHD, especially in pediatric cases, arises from their dual function as both defenders against infection and contributors to tissue damage [66]. The potential deleterious behavior of inflammasomes is likely to be due to a confluence of genetic predisposition, environmental influences, and the distinctive attributes of GAS [67,68]. Certain individuals may harbor genetic polymorphisms that increase sensitivity or hyperactive responses of inflammasomes to streptococcal antigens, resulting in an excessive inflammatory response and subsequent tissue damage. Inflammasome activation can be further intensified by environmental factors, such as repeated exposure to GAS, limited access to healthcare, or delayed treatment [30,69]. Moreover, GAS have developed diverse mechanisms to evade the immune system, potentially modulating inflammasome pathways in a manner that benefits their survival and promoting the establishment of a persistent inflammatory state.

The intricate interaction between host genetics, environmental factors, and bacterial evasion strategies can shift the equilibrium from a defensive immune response to a detrimental state of exaggerated activation of inflammasomes, thereby playing a role in the development and progression of RHD [54,70]. Moreover, in pediatric patients, this imbalance may cause further exacerbation due to the developing immune system’s unique responses to GAS infections, increasing the severity of inflammasome-mediated damage [71]. In summary, while inflammasomes are a vital component of immune responses to GAS infection, their continuous and uncontrolled activation within the framework of RHD can be detrimental, as they initiate the mechanisms that result in sustained cardiac injury. This is particularly concerning in children, in whom early and aggressive inflammasome behavior and activity can set the stage for lifelong cardiac complications. The investigation of this dysregulation and the exploration of strategies to regulate inflammasome activity hold the potential to mitigate the impact of this severe disease.

### 3.3. Kawasaki Disease

Kawasaki disease (KD), also known as mucocutaneous lymph node syndrome, is a unique and serious inflammatory condition that occurs predominantly in infants and young children under five years of age [72,73]. KD is the most common cause of acquired cardiac disease in the developed world, affecting blood vessels throughout the body, including the coronary arteries, leading to severe complications such as coronary artery lesions and endothelial cell (EC) dysfunction [74]. Usually self-limiting, KD first manifests with fever and is accompanied by other symptoms such as mucocutaneous inflammation and cervical adenopathy [74]. Although the exact etiology is unknown, it has been hypothesized to have a genetic, environmental, and immunological basis.

The genetic predisposition to KD has been detected in several epidemiological studies showing that, although seen across the world, KD is more common in Asian populations, especially Japanese children, in whom it is 10- to 15-times more common than in White children [72]. Supporting a genetic etiology, the incidence of KD in Japanese American children in Hawaii is the same as in Japan. Furthermore, the higher relative risk of KD appears to run in families, supporting a hereditary component of the disease.

Infectious agents, including *Streptococcus sanguis*, *Staphylococcus aureus*, *Yersinia pseudotuberculosis*, retroviruses, and Epstein–Barr virus [72] appear to act as triggers for KD. These pathogens can damage the coronary artery endothelial cells, leading to NLRP3 inflammasome-mediated pyroptosis, the latter playing a significant role in the development of KD [75,76,77,78]. Research supports the involvement of inflammasome activation in the pathogenesis of Kawasaki disease (KD). In vitro studies suggest that pyroptosis proteins contribute to the onset of KD and, in vivo studies indicate that inflammasome activation cytokines such as IL-1 and IL-18 are highly overexpressed in KD patients compared to healthy controls [75,79].

### 3.4. Ankylosing Spondylitis

A chronic inflammatory disease that mostly affects the axial skeleton and peripheral joints, ankylosing spondylitis (AS) causes the production of new bone [80]. There is a growing recognition of cardiovascular problems in AS patients, including aortitis, conduction abnormalities, and aortic regurgitation. Therefore, early intervention and monitoring are crucial [81]. AS inflammation is largely caused by macrophage inflammasome activation, specifically involving the NLRP3 inflammasome [82]. AS has been associated with elevated levels of caspase-1 and enhanced expression of the NLRP3 and ASC genes [83,84]. A possible connection between the microbiota and inflammasome activity is also suggested by the intestinal overexpression of NLRs and AIM2 [83]. Treatments that target IL-1, including anakinra, have demonstrated efficacy in controlling symptoms of AS [84].

The promotion of osteogenesis in AS is also linked to microRNA (miR)-21 activation through TNF-α-induced JAK2/STAT3 signalling, which further links inflammasome activation to the advancement of the disease [85]. Inflammasomes play a complex role in AS pathophysiology, as evidenced by the increased clarity surrounding these pathways.

### 3.5. Rheumatoid Arthritis

RA is a chronic autoimmune disease characterized by inflammation of the synovium in joints, leading to pain, swelling, and eventual joint damage. In RA, dysregulated immune responses activate various inflammatory pathways, including the classical and alternative complement pathways, as well as the production of pro-inflammatory cytokines such as TNF-α, IL-1, and IL-6 [86]. These cytokines play pivotal roles in orchestrating the inflammatory cascade within the synovium, perpetuating synovial hyperplasia and cartilage destruction [87]. RA is not only a disease of joints but a multi-system disorder that also significantly impacts cardiovascular health, leading to increased morbidity and mortality from CVDs. Chronic systemic inflammation associated with RA contributes to accelerated atherosclerosis, endothelial dysfunction, and increased risk of MI, stroke, and heart failure [88].

Against this background, NLRP3 inflammasomes have emerged as a key mediator of inflammation in RA. The activation of NLRP3 inflammasomes has been implicated in driving joint inflammation and destruction. For example, elevated levels of NLRP3 inflammasome components have been detected in the synovial fluid of RA patients [89]. Furthermore, preclinical studies have demonstrated that pharmacological inhibition of NLRP3 attenuates disease severity in animal models of RA, highlighting the therapeutic potential of targeting NLRP3 inflammasome activation to treat RA.

One interesting player in inflammasome pathobiology is the transcription factor nuclear factor erythroid 2-related factor 2 (NRF2). NRF2 is a redox regulator that has antioxidant and anti-inflammatory roles, and was shown to downregulate NLRP3 [90]. In RA, NRF2 activation protects chondrocytes (cartilage cells) and osteoblasts (bone-forming cells) from oxidative stress and inflammation-induced apoptosis [83]. Interestingly, the therapeutic value of targeting NRF2 is further supported by promising NRF2 activators currently at various stages of drug development. For example, RTA 408 (omaveloxolone) has been found to attenuate osteoclastogenesis by inhibiting NF-κB signaling [55]. Another synthetic component is bardoxolone methyl (CDDO-Me), which has been shown to break the vicious cycle between M1 macrophages and senescent nucleus pulposus cells through the NF-κB pathway [91]. In addition to these synthetic compounds, some natural compounds have also been shown to activate NRF2 such as sulforaphane, curcumin, and epigallocatechin-3-gallate [92,93,94].

**Table 1:** Summary of inflammasome-mediated pathology in cardio-rheumatic diseases.

Disease	Inflammasome/Related Protein and Pathway	References
Familial Mediterranean fever	Pyrin and IL-1β: FMF is characterized by mutations in the *MEFV* gene encoding pyrin. These mutations result in a defective pyrin protein that cannot properly regulate IL-1β production, leading to excessive inflammation and episodes of fever. Pyrin dysfunction is a primary driver of the disease’s autoinflammatory episodes.	[95,96]
Rheumatic heart disease	NLRP3 inflammasome and *Streptococcus*-triggered responses: in RHD, the immune response to *Streptococcus* bacteria can aberrantly activate NLRP3 inflammasomes. This activation contributes to inflammation and subsequent damage to heart valves.	[59,97]
Kawasaki disease	NLRP3 inflammasome: this complex plays a critical role in activating IL-1β, a pro-inflammatory cytokine. Its activation in Kawasaki disease is associated with the severe inflammation seen in blood vessels, which is central to the disease’s pathology.	[78,79]
Ankylosing spondylitis	NLRP3, IL-1β, and IL-18: ankylosing spondylitis is associated with dysregulation of the inflammasome pathway, particularly the NLRP3 inflammasome, leading to increased production of pro-inflammatory cytokines such as interleukin-1β and IL-18, contributing to the chronic inflammation and spinal involvement characteristic of the disease. Targeting the inflammasome pathway is a focus of research for potential therapeutic interventions in ankylosing spondylitis.	[52,53,56]
Rheumatoid arthritis	Immune dysregulation in RA activates inflammatory pathways and pro-inflammatory cytokines like TNF-α, IL-1, and IL-6, causing synovial hyperplasia and cartilage destruction. RA also impacts cardiovascular health, increasing the risk of CVDs due to systemic inflammation. The NLRP3 inflammasome is a key mediator in RA inflammation, and inhibiting it reduces disease severity. NRF2, a transcription factor, downregulates NLRP3 and protects joint cells from oxidative stress. Promising NRF2 activators like RTA 408, bardoxolone methyl, sulforaphane, curcumin, and EGCG are in development.	[83,90,91,92,93,94]

## 4. Future Directions

Given the importance of establishing the pathways underlying cardio-rheumatic diseases, there is a need for accurate models that mimic human disease states. The use of human induced pluripotent stem cell-derived cardiomyocytes (hiPSC-CMs) is expected to become more frequent, as they are useful for replicating cardiac diseases and can be specific to each patient, facilitating the creation of personalized approaches for these conditions [80]. hiPSC-CMs will be especially useful for investigating variants in inflammasome genes that alter disease susceptibility and severity. The field is also expected to benefit from improvements and advances in gene editing technologies such as CRISPR/Cas9, enabling more advanced and sophisticated models of heart diseases and exploring the therapeutic potential of targeting specific inflammasome components. Gene editing also provides the opportunity to correct mutations in cells acquired from patients, again facilitating the personalized medicine approach.

Moreover, recent advances in novel therapeutics that specifically target inflammasomes are showing promise, and a number of small molecule inhibitors and biologics targeting inflammasomes are currently being examined. These therapeutics are exciting candidates for heart diseases, as they not only reduce inflammation but also prevent fibrosis, thereby enhancing heart function. Mechanistic studies on inflammasome function in cardiac disease are likely to benefit from integrating advanced computational modelling and imaging techniques. Implementing these tools will facilitate more accurate mapping of inflammasome activity and how it influences cardiac function [81,82]. Finally, future research is expected to include larger-scale genetic backgrounds and environmental factors, ensuring the generalizability of the findings and accurate customization of treatments to specific populations.

## 5. Conclusions

Inflammasomes are a fundamental component of the innate immune system in response to the detection of pathogens or injury. However, after prolonged activation, they can transition from being helpful to harmful, potentially triggering CAD. Development of therapies to target dysregulated NLRP3 activity and the IL-1 production driving the pathogenesis of these diseases. 

## Data Availability

Not applicable.

## Abbreviations

The following abbreviations are used in this manuscript:

ALR	Absent In Melanoma 2-Like Receptor
ARF	Acute Rheumatic Fever
AS	Ankylosing Spondylitis
CAD	Coronary Artery Disease
CVD	Cardiovascular Disease
CARD	Caspase Activation and Recruitment Domain
DAMP	Damage-Associated Molecular Patterns
EC	Endothelial Cell
FMF	Familial Mediterranean Fever
IFN	Interferon
IL	Interleukin
KD	Kawasaki Disease
miR	microRNA
MI	Myocardial Infarction
NLR	Nucleotide-Binding Domain-Like Receptor
NF	Nuclear Factor
NRF2	Nuclear Factor Erythroid 2-Related Factor 2
NK	Natural Killer
oxLDL	Oxidized Low-Density Lipoprotein
PAMP	Pathogen-Associated Molecular Patterns
PRR	Pattern Recognition Receptor
ROS	Reactive Oxygen Species
RHD	Rheumatic Heart Disease
RA	Rheumatoid Arthritis
SLE	Systemic Lupus Erythematosus
TLR	Toll-Like Receptors
